# Is Common Dandelion (*Taraxacum officinale* agg.) Foraged for Food in Vineyards Pesticide Residues Free?

**DOI:** 10.3390/foods14040684

**Published:** 2025-02-17

**Authors:** Maruša Skubic, Helena Baša Česnik, Špela Velikonja Bolta, Denis Rusjan, Helena Šircelj

**Affiliations:** 1 Department of Agronomy, Biotechnical Faculty, University of Ljubljana, Jamnikarjeva ulica 101, 1000 Ljubljana, Slovenia; helena.sircelj@bf.uni-lj.si (H.Š.); denis.rusjan@bf.uni-lj.si (D.R.); 2 Central Laboratories, Agricultural Institute of Slovenia, Hacquetova ulica 17, 1000 Ljubljana, Slovenia; helena.basa-cesnik@kis.si (H.B.Č.); spela.velikonja-bolta@kis.si (Š.V.B.)

**Keywords:** common dandelion, *Taraxacum officinale* agg., pesticide residues, fungicides, vineyards, wild edible plants, food safety, risk assessment

## Abstract

Consumption of common dandelion (*Taraxacum officinale* agg.) can pose a risk when foraged in agroecosystems like vineyards where pesticides are frequently used. The aims of our study are to evaluate whether dandelion foraged in vineyards with different management practices (integrated pest management, organic, and biodynamic) in spring is suitable for consumption and to assess whether the contents of selected pesticide residues (PR) in integrated vineyards in dandelion vary throughout the seasons. Young dandelion leaves were sampled in spring, summer, and autumn in integrated vineyards, while in spring also in organic and biodynamic vineyards. The selected PR was analyzed using GC-MS/MS and LC-MS/MS, using extraction with acetonitrile. The method was validated on a dandelion matrix according to international guideline SANTE 11312/2021. Despite the use of many pesticides in integrated vineyards, only tebuconazole was detected in spring in one sample (0.005 mg/kg), while no PR was detected in dandelion from organic and biodynamic vineyards. However, at summer sampling seven different PR were detected, of which the kresoxim-methyl maximum residue limit was exceeded in five samples (0.012–0.029 mg/kg), while in autumn no PR was detected. Based on this study, it seems that dandelion leaves foraged in vineyards in spring could be unproblematic for consumption.

## 1. Introduction

Since ancient times, people gathered wild plants for food all over the world. Many species which are now considered as undesired weeds were ancient cultures’ main source of food [1]. In recent years, there has been an increased interest in foraging and consuming wild edible plants in Europe, probably because of awareness, that many wild plant species contain health-promoting bioactive substances [1,2,3]. Despite many positive effects, foraging can represent a threat if wild plants are foraged in intensive agricultural areas.

Due to the growing need for food, agriculture has been considerably intensified in the last 60–80 years, and since then many different pesticides have been in use. According to data from the 2023 FAOSTAT, around 350,000 tons of pesticides were sold in the European Union per year [4]. Among them, fungicides and bactericides were the most sold group of pesticides in 2021 [4]. The use of pesticides has many negative impacts on the quality of air [5] and water [6], herbicides use could cause soil erosion [7], and pesticides reduce biodiversity in the surrounding area as they affect non-target organisms [8]. They also represent a major threat to human health, as their residues in food are neurotoxic, disrupt the immune and endocrine system, and are mutagenic and carcinogenic [9,10,11]. Therefore, the quality and safety of foraged food can be diminished in case of consuming plants from areas with high use of different pesticides.

Viticulture is in agriculture the biggest fungicide user in many European countries [12,13]. In the European Food Safety Authority report [14] pesticide residues were detected in 81.7% of grapes. This is probably a consequence of the fact, that most worldwide cultivated grapevine (*Vitis vinifera* L.) varieties are highly susceptible to fungal diseases [15], which can cause significant economic loss [16,17]. For many years, the European Union has been trying to reduce the use of pesticides in food production [18,19], but the transitions in viticulture are often very slow reflecting also just a moderate reduction of synthetic fungicide uses. Also in Slovenia, since the 1990s, we have started to introduce additional restrictions and controls on the use of pesticides in viticulture, and to date we have greatly reduced the number of authorized products and the number of their applications. The share of organic grape production in Slovenia has increased to 5.8%, while integrated pest and disease management still present the main grape production [20].

In integrated pest and disease management (IPM) in Slovenian vineyards, many synthetic pesticides are allowed, but their use is highly controlled and limited [21]. On the other hand, in organic production (ORG) most synthetic pesticides are forbidden, only copper and sulfur-based fungicides are allowed [19,22]. In biodynamically managed vineyards (BIOD), no artificially synthesized pesticides are allowed; instead, a series of soil and plant amendments called preparations are used [23]. Despite strict regulations about pesticide use in ORG and BIOD-managed vineyards, some pesticide residues can be detected also there due to cross-contamination from the surrounding area or some other reasons [24,25].

Studies on the use of pesticides in vineyards mainly focus on pesticide residues in grapes and wines [24,25] as well as in the soil [26,27], and there are few publications on pesticide residues in the leaves of the vine [28,29,30]. But to the best of our knowledge and insight into the scientific literature, studies of pesticide residues in edible plants such as common dandelion foraged in vineyards have not been conducted yet. This is surprising since it is about human nutrition, which has a significant impact on health [9,10,11].

The herbaceous perennial common dandelion (*Taraxacum officinale* agg.) is occurring worldwide in temperate regions including agricultural areas such as vineyards, where different pesticides are in use [31]. It is not growing just in the wild, but it has been also cultivated for food [32]. Traditional consumption has been reported for millennia in many Mediterranean countries [33,34,35,36,37]. Dandelion is used for food, it can be used in ravioli or quiche filling, in omelets, risotto, as cooked or fried vegetables, and leaves can be eaten raw in salads [3,38]. The rosettes of young dandelion leaves are best to collect and use in spring between March and May, before flowering, after that only the upper part of the leaves are palatable enough to be eaten fresh [38].

Due to its common use for culinary and medicinal purposes, it is important that foraged dandelion plants do not contain any chemical contaminants which could negatively affect human health. Few studies about chemical pollutants in dandelion are available. In aerial parts of dandelion, polychlorinated biphenyls (PCBs) were found [39] and in commercial herbal teas of dandelion, several polycyclic aromatic hydrocarbons (PAHs) were detected [40]. Some insecticides were detected in dandelion flowers [41], while in the study of Boguś et al. [42], where dandelion samples were taken from meadows and urban areas, no pesticides were detected in roots, leaves, or flowers. Not only, that on so frequently foraged plants only a few studies about pesticide residues were made, but to the best of our knowledge, there were no studies of pesticide residues in dandelion growing in vineyards at all, despite being often foraged in vineyards.

Therefore, the aims of our study were to evaluate the presence of selected pesticides in dandelion leaves (i) foraged in spring in vineyards under different management practices (integrated pest management, organic and biodynamic grape production) and (ii) foraged also in summer and autumn in vineyards under IPM, where spraying with pesticides is still most frequent and presence of residues as well. With a complex and broad topic of interest to study, we will report, for the first time, data on pesticide residues in edible common dandelion leaves foraged seasonally in intensive agricultural areas, thereby contributing to the science of the harmfulness of this type of foraging and its potential impact on human health.

## 2. Materials and Methods

### 2.1. Sampling

Rosettes of young tender leaves of common dandelion (*T. officinale* agg.) growing in an interrow space in five vineyards under IPM from Goriška brda, a traditional intensive winegrowing area in the western part of Slovenia, were sampled during the season, first in spring (28 and 29 March), second in summer (4 and 5 July) when the plants regrew after the mulching, and third in autumn (5 and 6 October) when the plants regrew again after the last mulching preceding grape harvest. Moreover, the vineyards have been included in IPM for at least 15 years, and their production is controlled by the certified control organization KON-CERT, located in Maribor, Slovenia. In spring, dandelion from an additional five vineyards under ORG and four vineyards under BIOD grape production was sampled. ORG vineyards have been under organic production for at least 7 years, while those under BIOD for at least 12 years, are controlled by certified control organizations KON-CERT and DEMETER, respectively. At the time of sampling, all certificates were up to date. The climate in Goriška brda is sub-Mediterranean, where the average annual temperature of the last 10 years is around 13 °C and the average rainfall 1600 mm [43]. The soil in the vineyards is quite homogeneous, eutric brown soil type on flysch dominates in most of the terraced vineyards where dandelion was sampled [44].

Two pooled samples per vineyard were collected (each vineyard was divided into two halves, on each half one pooled sample was collected). One pooled sample consisted of several rosettes of young leaves collected in every second row of one half of the vineyard. Rosettes were about the same size, cut with a knife just above the root, and collected alternately in the middle of the row and the next one under the vine in zik-zak line. Around 150 g of dandelion leaves were collected in three liters of plastic bags, stored in and lately transported in a portable refrigerator to a laboratory, where yellow, damaged, or with soil covered leaves were removed as stated in Commission regulation 2018/62 for witloof group, where dandelion belongs [45]. Unwashed fresh dandelion material was then mixed and stirred with a food processor and kept in plastic vessels at −20 °C until extraction. Dandelion leaves were unwashed according to the recommendations for analytical methods of pesticide residues, where the raw plant is analyzed [46]. Altogether, 48 dandelion samples were collected, 28 in spring (March), 10 in summer (July), and 10 in autumn (October).

### 2.2. Chemicals

Standards of boscalid, difenoconazole, dimethomorph, kresoxim-methyl, mandipropamid, metalaxyl-M, tebuconazole and triadimenol were purchased from Dr. Ehrenstorfer (LGC Labor GmbH, Augsburg, Germany). Acetone and methanol were from Honeywell Building Solutions (Hamburg, Germnay), acetonitrile was from J. T. Baker (Gliwice, Poland) and anhydrous sodium sulfate (Na_2_SO_4_) was from Merck KGaA (Darmstadt, Germany).

### 2.3. Extraction and Clean-Up of Pesticide Residues

To 10 g of mixed samples, 15 mL of acetonitrile and 5 g of anhydrous Na_2_SO_4_ were added, homogenized with an Ultra-Turrax T25 (Ika-Werke, Staufen, Germany) for two minutes and filtered through filter paper black strip (Sartorius Lab Instruments GmbH, Goettingen, Germany) filled with 10 g of anhydrous Na_2_SO_4_. Notably, 1.5 mL of that extract was added to previously conditioned column Superclean Ultra 2400 3 mL (Supelco, Sigma-Aldrich, St. Louis, MO, USA), then rinsed with 16 mL of acetonitrile. The eluent was evaporated on a rotary evaporator (Büchi Rotavapor R-114, Flawil, Switzerland) and blown to dryness under nitrogen. To dry eluent, 1 mL of acetone was added, and dissolved in ultrasonic bath of Asonic PRO 30 (ASonic, Shanghai, China), the solution was transferred to vial and blown to dryness under nitrogen, then 200 μL of acetone for HPLC was added. So prepared extract was further analyzed with gas chromatograph (Agilent Technologies 8890, Shanghai, China) coupled with tandem mass spectrometer (Agilent Technologies 7010B, Santa Clara, CA, USA) (GC-MS/MS).

Moreover, 100 μL of final extract was transferred to another vial, blown to dryness under nitrogen, and dissolved with 100 μL of methanol for HPLC. This extract was further analyzed with liquid chromatograph (Agilent Infinity 1290, Palo Alto, CA, USA) coupled with tandem mass spectrometer (Agilent Technologies Palo Alto, CA, USA) (LC-MS/MS). The extraction was optimized in this study.

### 2.4. Determination of Pesticide Residues

**Figure 1:**
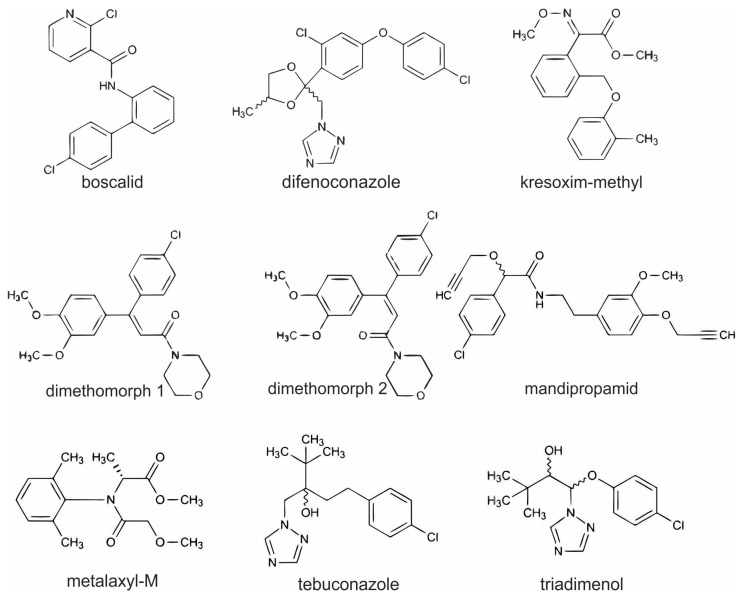
Chemical structures of analyzed pesticide residuals in dandelions from vineyards (chemical structures are taken and rearranged from Lewis et al. [48]).

The selection of active substances for the analysis of dandelion leaves was based on the list of pesticides used in selected vineyards with integrated management that were included in our study. The fungicides used in the year before sampling and in the year of sampling are listed in Supplementary Materials Tables S1 and S2. From the list of fungicides used in the years prior to sampling in sampled vineyards, we have selected those that are most commonly used in the surrounding area and that could potentially affect human health. One pesticide (triadimenol) was included in our study to check whether its use had been discontinued. It was commonly used in these vineyards before 2021, but its use has been banned in Slovenia since February 2021 [47]. Other analyzed pesticides were used in a year before sampling and in a year after sampling. Eight active substances were analyzed using two multiresidual methods, their chemical structures are represented in Figure 1. Analysis methods were optimized in this study. Boscalid, kresoxim-methyl, metalaxyl-M, tebuconazole, and triadimenol were analyzed with GC-MS/MS. The compounds were separated into an Agilent J & W, HP-5MS UI column (30 m × 0.25 mm ID, 0.25 μm; Agilent Technologies, CA, USA) at a constant flow of helium at 1.2 mL min^−1^ The GC oven was programmed as follows: 55 °C for 2 min, from 55 °C to 180 °C at 25 °C min^−1^, at 180 °C for 20 min, from 180 °C to 280 °C at 20 °C min^−1^, at 280 °C for 20 min. The temperature of the ion source was 230 °C, the auxiliary temperature was 280 °C and the quadrupole temperature was 150 °C. For qualitative and quantitative determination, the MRM transitions were used presented in Table 1. For each active substance, two transitions were scanned. For the calibration matrix, match standards were used.

**Figure 2:**
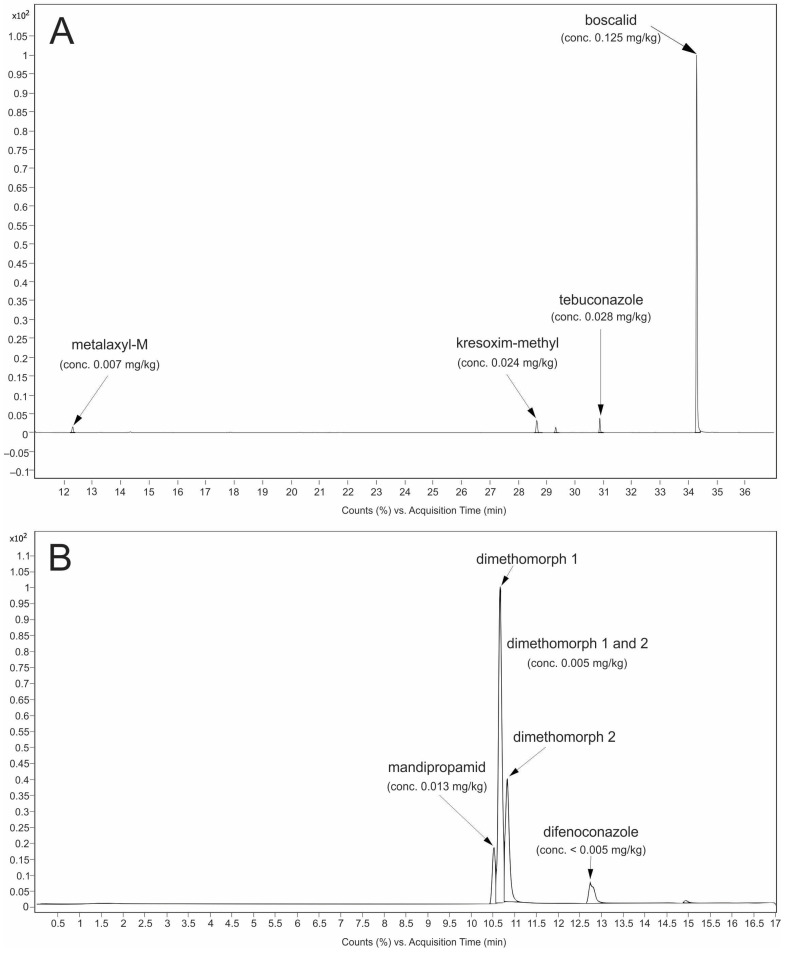
(**A**) Chromatogram of some pesticide residuals and its concentrations detected on GC-MS/MS; (**B**) Chromatogram of some pesticide residuals and its concentrations detected on LC-MS/MS.

Difenoconazole, dimethomorph, and mandipropamid were analyzed with LC-MS/MS in ESI+ mode, using a Supelco Titan C18 column (10 cm × 2.1 mm, 1.9 µm; Supelco, MA, USA) for separation. The flow was 0.3 mL min^−1^ and the gradient was as follows: start at 30% B and hold for 5 min, increase to 90% B in 15 min, then increase to 100 B in 16 min, hold 100% B for 5 min, decrease to 30% B in 1 min, post-run 3 min at 30% B. Source temperature was 250 °C, gas flow 6 L min^−1^, sheath gas flow 10 L min^−1^, sheath gas temperature 375 °C and nebulizer pressure 35 psi. Quadrupole temperatures were 100 °C. Each sample was injected once in ESI+ mode. For each compound, two transitions were monitored, hence the fragmentor and collision cell voltage were optimized. MRM transitions are presented in Table 1. For the calibration matrix, match standards were used. Chromatograms of some of the detected pesticide residuals in our samples can be seen in Figure 2.

### 2.5. Method Validation

The multiresidue method was validated on dandelion matrix for all selected pesticides according to SANTE 11312/2021 [49]. In the Table 2 limit of quantification (LOQ), linearity range, recovery with belonging relative standard deviation, uncertainty of repeatability and reproducibility are shown.

The linearity was verified using the matrix match standards (two repetitions for one concentration level, 9 to 14 concentration levels for the calibration curve). The linearity and range were determined by linear regression, using the F test. R2 varied between 0.993 to 0.999.

LOQs were estimated from the chromatograms of matrix match standards. LOQs were chosen at a minimum of S/N = 10. LOQ for all substances was 0.005 mg kg^−1^.

#### 2.5.1. Precision

Blank dandelion was sampled and analyzed to prove that it contains no pesticide residues. For the determination of precision according to ISO 5725 [50], i.e., repeatability and reproducibility, the extracts of spiked blank dandelion were analyzed at LOQ. Within a period of 10 days, two parallel extracts were prepared each day for each concentration level. Each one was injected once. Then the standard deviation of the repeatability of the level and the standard deviation of reproducibility of the level were both calculated.

#### 2.5.2. Uncertainty of Repeatability and Uncertainty of Reproducibility

The uncertainty of repeatability and the uncertainty of reproducibility were calculated by multiplying the standard deviation of repeatability and the standard deviation of reproducibility by the Student’s *t* factor, for nine degrees of freedom and a 95% confidence level (t_95;9_ = 2.262).


Ur = t_95;9_ × S_r_; U_R_ = t_95;9_ × S_R_

Highest measurement uncertainty of repeatability was 20.0% and highest measurement uncertainty of reproducibility was 34.0%. The measurement uncertainty for PPP residues should be ≤50%, as proposed in SANTE/11312/2021 [49]. During validation it was proven that measurement uncertainty is below required 50%.

#### 2.5.3. Accuracy

The accuracy was verified by checking the recoveries. The average of the recoveries from the tests for precision (10 days, 2 parallel samples each day) was calculated. According to the requirements for method validation procedures SANTE [49], acceptable mean recoveries are those within the range of 70% to 120%, with associated repeatability of RSDr ≤ 20%.

According to the guidelines for single-laboratory validation [51], acceptable mean recoveries at level > 0.001 mg kg^−1^ ≤ 0.01 mg kg^−1^ are those within the range of 60% to 120%, with an associated repeatability RSDr ≤ 30%.

Our recoveries ranged from 71.5 to 85.0% with RSDs 5.9 to 17.7%, meaning that they are in accordance with SANTE [49] and Alder et al. [51].

### 2.6. Risk Assessment

For residues of kresoxim-methyl exceeding maximum residue limit (MRL) short-term exposure was calculated using the EFSA PRIMo model revision 3.1 [52]. Input value was the Highest residue (HR) and since Acute Reference Dose (ARfD) was not set, as worst-case situation Acceptable Daily Intake (ADI) was used instead. Acute consumer exposure was expressed in % of the ADI. The acceptable limit for short-term exposure is 100% of the ADI.

## 3. Results and Discussion

Dandelion is one of the first wild edible plants in spring which is used in many recipes, but its consumption could be potentially dangerous if it is grown and harvested in an intensive agricultural area, such as vineyards, which are known to be one of the most pesticide using areas [13]. According to the Technological instructions for integrated grape production [21] more than 40 different fungicides are allowed to be used in vineyards against main diseases such as downy mildew, powdery mildew, gray mold, black rot, etc., use of each depends on the year regarding the weather conditions and diseases pressure. In selected integrated vineyards of Goriška brda 20 different active substances of fungicides were used in a year of sampling (Supplementary Materials Table S1), among them seven commonly used fungicides were included in our study and one more pesticide, which have been used two years before sampling and it is forbidden since then. Spraying started at the end of March and the last application was at the end of July (Supplementary Materials Table S2).

In our study, none of the analyzed pesticide residues were found in 96.4% of all dandelion leaf samples collected in spring in vineyards with different management practices. In spring 2022, in only one sample of dandelion leaves from IPM vineyard, tebuconazole was detected, whose content was at its limit of quantification (0.005 mg kg^−1^—Table 2). Tebuconazole is the active substance in eight fungicides that are allowed in IPM against powdery mildew and their use is limited to a maximum of three times in one season [21], due to its good performance, fungicides with this active substance are recommended for protection in the late spring and early summer months when the climate conditions are suitable for grape infections with powdery mildew. Previously it was already detected in wine [53], in grapes [54], and in other fruits such as mandarins, nectarines, peaches, plums, and lemons [54,55] and in vegetables as peas, peppers, tomatoes, carrots, etc. [54,55]. Tebuconazole is known as potentially dangerous, because it affects reproduction through malformations and post-implantation loss, disrupts the endocrine system, and affects the liver [56]. Tebuconazole has, based on the data from the Pesticide Properties DataBase [48], on average the longest dissipation rate RL50 (9.9 days) in plant matrix among all analyzed active substances from our study. Based on the data from the Pesticide Properties DataBase [48] the RL50 for tebuconazole can be between 1.0–59.4 days, while in another study where RL50 was assessed in grapes, it ranged between 5.3 and 17.4 days [57]. Different data for tebuconazole’s RL50 are probably the consequence of the fact that dissipation rates for many pesticides depend on many factors including time of application, spraying concentration, environmental factors, location, etc. [57,58]. The presence of tebuconazole in dandelion leaves sampled in spring 2022 could be explained by the use of fungicide based on tebuconazole in the previous year 2021 in July, which at the same time, was not expected, as the manufacturer’s instructions regarding the withdrawal period reports 14–35 days [21]. Moreover, the rather dry autumn and winter of 2021 [43] might also contribute to the presence of tebuconazole in the dandelion leaves in spring. A higher amount of precipitation would increase the leaching of the pesticides from leaves [59,60,61], but due to rather dry weather in our case, tebuconazole could stay on the plant for a longer period. However, one of eight studied PRs was detected in dandelion leaves collected in spring in one of the five IPM vineyards, in which MRL was far below the values reported as critical for human health. Therefore, we can conclude that dandelion leaves foraged in spring could be safe for consumption also from IPM vineyards.

In dandelion leaves from ORG and BIOD vineyards no active compounds were detected at all (Supplementary Materials Table S3), which was to some extent expected as none of these fungicides are allowed in both grape production [22,23], although certain deviations are possible, mainly due to the fact that the vineyards in Goriška brda district are quite small (in average 0.6 ha per vineyard; [20]) and there could be contamination of plants from spraying in neighboring vineyard under IPM, due to spray drift, which is common in vineyards and in other agricultural areas [62,63]. Moreover, some studies reported the presence of pesticides also in organic wines, supposedly grape contaminated by spraying in nearby vineyards [24,25]. Once more, based on our results, dandelion leaves foraged in spring from ORG and BIOD vineyards were free of pesticides and therefore supposedly safe for consumption.

**Figure 3:**
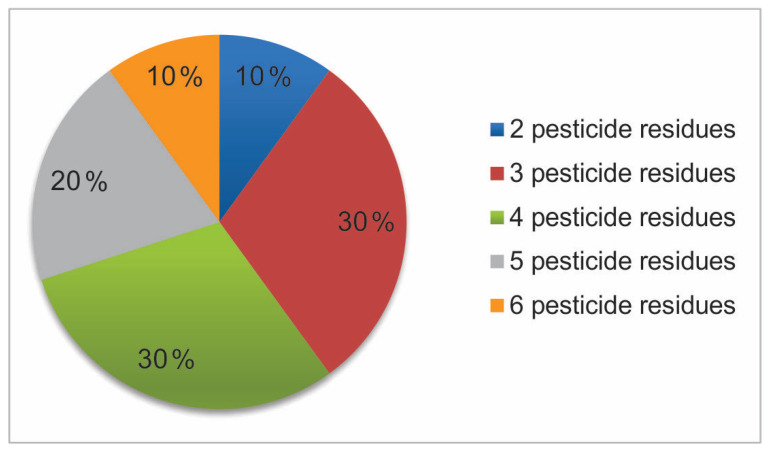
Percentages of dandelion samples from the summer sampling with multiple pesticide residues detected.

**Figure 4:**
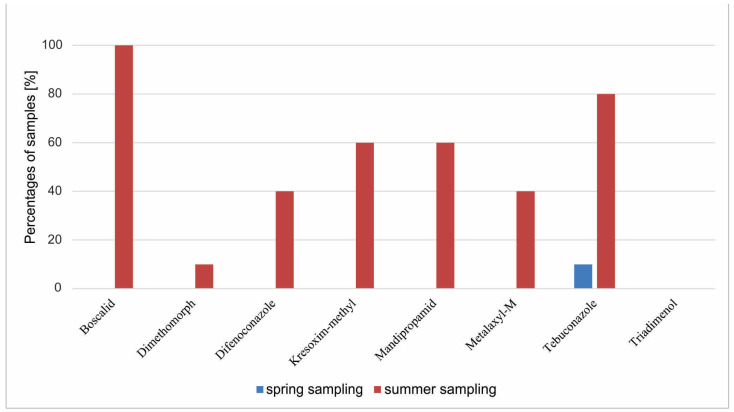
Frequencies of detected selected pesticide residues in dandelion samples in spring and summer sampling from vineyards with integrated pest management.

In summer and autumn, sampling only dandelion from IPM vineyards was collected. In summer, two to six active substances were identified in each sample of dandelion leaves from IPM vineyards (Figure 3). Percentages of samples containing one or more PR are seen in Figure 3. Frequencies and ranges of detected selected active substances in dandelion samples from integrated vineyards are presented in Figure 4 and Table 3. The most frequently of summer identified active substance was boscalid, which was detected in all samples of dandelion leaves. The second most frequently found active substance was tebuconazole (found in eight out of 10 samples). The third most frequently found active substances in summer sampling were kresoxim-methyl and mandipropamid, both found in six samples (60% of samples from second sampling), followed by metalaxyl-M and difenoconazole found in four samples (40% of samples from second sampling). The least frequently found PR in dandelion from integrated vineyards in summer sampling was dimethomorph, found in one sample (10% of samples from summer sampling). All of these detected PR could affect human health. According to expectations, triadimenol was under the limit of quantification in all analyzed samples, as stocks of product Falcon EC 460 which besides tebukonazol and spiroksamin contain as well active substance triadimenol had to be used by February 2021, and since then, it has been abandoned [47]. No other registered and permitted products containing triadimenol have been allowed in grape production in Slovenia since then [64].

Boscalid is an active substance in only two of the permitted fungicides against powdery mildew and gray mold in IPM grape production, and its presence in the summer leaves of dandelion was expected, as a systemic fungicide based on boscalid is usually used in the vine phenophase when the majority of berries on the bunch are touching (BBCH 79). Boscalid appears to be one of the most persistent pesticides in grapes during ripening [65]. It was detected in wine samples of conventional production [24,53], in grapes [55], and also in apples [66] and vegetables such as pepper, lettuce, and tomato [55]. Boscalid affects the liver and thyroid; it shows reproductive toxicity as it causes delayed ossification of offspring reduces viability and decreases pup body weight during lactation [67]. Moreover, in the same summer leaves of dandelion, the active substance kresoxim-methyl was also identified as the third most identified PR. Its detection confirms the use of the systemic fungicide against powdery mildew. Kresoxim-methyl is potentially carcinogenic; it causes delayed development of pups and is toxic to the liver [68,69]. It has already been detected in grapes [54,55,70], currants, strawberries [54], and in samples of pepper and zucchini [54,55].

In our study, the third most frequently identified active substance in summer dandelion leaves, besides kresoxim-methyl, was mandipropamid, which is present in six systemic fungicides against downy mildew allowed in IPM vineyards. In 2022, the weather conditions during late spring and early summer in the Goriška Brda winegrowing district were quite humid, which increased the risk of downy mildew infections, and to prevent them, systemic fungicides were used frequently. It was already detected in wine [24]. Mandipropamid is toxic to the liver, as well as metalaxyl-M [71,72], which is an active substance in only one of the systemic fungicides allowed in IPM grape production against downy mildew and whose use is limited to a maximum of three times per season [21]. There are some reports on metalaxyl-M in grapes [73], in wine [24,53]; beside that, it was found in several vegetables such as cucumbers, potatoes, and tomatoes [55], which shows that it is used as a fungicide for various crops.

Difenoconazole and metalaxyl-M were identified in 40% of summer dandelion leaves collected in IPM vineyards in our study. Difenoconazole is the main active agent in four systemic fungicides allowed in IPM grape production against powdery mildew, the use of which is limited to a maximum of twice per season due to the possibility of resistance [21]. According to the mode of action, the use of fungicides based on difenoconazole is recommended only when the risk of infection with powdery mildew is high; therefore, most spraying with these fungicides is done in late spring and early summer during hot days. It has previously been found in grapes [54], pears, apples, papayas, carambola, pitayas [54,55], as well as in carrots, celery, tomatoes, and beans [54]. Difenoconazole causes liver carcinomas in mice at high doses but is considered unlikely to represent a carcinogenic risk to humans; it is toxic to the liver, heart, thyroid, and kidneys [74]. Dimethomorph, which was found only in one summer dandelion sample, is present in two systemic fungicides against downy mildew and can be used a maximum of three times per season only in vineyards registered for wine production [21]. It has already been detected in wine [24,53,54] and grapes [54,73], as well as in tangerines, oranges, melons, cucumbers, lettuce, and tomatoes [54,55]. It could have toxic effects on the liver and prostate [75].

As mentioned before, pesticide residues were found in all summer dandelion samples, while in spring and autumn samples from IPM vineyards, there were almost no detected PR (except tebuconazole in spring, Table 3). These results are probably the consequence of integrated vineyard management. Spraying in integrated vineyards in Goriška Brda usually starts in May and ends in July; until the middle of August, only copper and sulfur-based fungicides are in use [21] (Table S1, Table S2). Our summer sampling was conducted just after the most intensive spraying period in integrated vineyards, which was the most probable reason why most pesticides were detected in dandelion leaves collected in summer from interrow spaces.

There is no available publication on PR in dandelion from vineyards to be used for comparison with the results from our study. On the other hand, some reports on PR in edible leaves of vine (Vitis vinifera L.) have been published [28,29,30]. Vine leaves are collected for culinary purposes in summer in several Mediterranean countries; they can be consumed fresh or in brine [30]. If spraying grapes leads to PR on the leaves of the vines, this might also affect plants, such as dandelion, growing underneath or in the vicinity. As observed in dandelion leaves collected in summer from our study, several different pesticide residues were also found in vine leaves in other studies [28,29,30]. However, the main difference between the results of our study and the aforementioned studies is how often European Union (EU) Maximum Residue Levels (MRL) were exceeded. In the study by El Din et al. [28], MRL values were exceeded for all detected PR, while in the studies by Hamzawy [29] and Zorlu Ünlü et al. [30], MRL values were exceeded for more than half of the detected PR, contrary to our study, in which the MRL value was exceeded for only one PR (Table 4). El Din et al. [28] in his study suggest that such high rates of PR in Egyptian vine leaves could be the consequence of pesticide misuse, overuse, or application of different types of pesticides to protect the vine. He also highlighted that there had been some recommendations for the use of PR on grapes by the Egyptian Ministry of Agriculture, but that they lacked proper regulations on a pesticide monitoring program [28].

In Table 4, sample portions below the limit of quantification and above MRL values, as determined by European Union legislation [76], from IPM vineyards in three sampling periods are presented. The only active substance in which dandelion samples exceeded MRL in our study was the potentially carcinogenic kresoxim-methyl from summer sampling [68,69]. It exceeded MRL in four samples (40% of samples from the second sampling); in two samples, it was above the limit of quantification but below MRL. A short-term risk assessment was conducted for those samples that exceeded the valid MRL of 0.01 mg kg^−1^. The highest short-term exposure represented 0.3% of the Acceptable Daily Intake (ADI) for children consuming dandelion, meaning that despite the fact that residues exceeded MRL, dandelion was safe for consumers.

The third sampling of dandelion leaves in IPM vineyards occurred at the beginning of October, at least two weeks after grape harvest and two months after the last fungicide application on the vines. No PR was identified in any sample of dandelion leaves collected at that time in IPM vineyards. There were no studies of PR in dandelion, nor in vine leaves collected in autumn; however, several studies from Slovenia and neighboring Croatia, contrary to our studies, reported pesticide residues in grapes and wine [24,53,70,73]. This could be explained by the fact that the spraying is largely limited to the leaf canopy of the vine, that the losses of fungicides to the surroundings are minor, and that under different weather conditions, PR on surrounding plants are washed from them [60,61], volatilized, degraded by microorganisms, photolyzed, or by other mechanisms [77]. Another possible explanation for the absence of pesticides in the dandelion samples collected in autumn may be attributed to the youth of the leaves, which regrew after the mulching in August when fungicide spraying in vineyards ceased. It appears that the plants either did not uptake PR from the soil or that the microorganisms in the soil already degraded them [78]. But in the case of grapes (and consequently wine) from the above-mentioned studies, these grapes collected at harvest are the same as at the time when the most intensive spraying occurred.

## 4. Conclusions

Based on our data, consuming dandelion leaves collected in vineyards in the European Union, which is a common practice in winegrowing regions, especially in spring, is relatively safe from the point of view of PR content. At that time, there was no concern while consuming dandelion collected in ORG and BIOD vineyards. In IPM vineyards, only one of eight analyzed pesticides in only one sample (out of 10) at its limit of quantification was identified. Due to its low concentration, below MRL value, it does not represent a threat to human health. But, of course, broader sampling and analysis of other potentially used pesticides in vineyards would be appreciated to confirm that dandelion leaves could be safe for consumption when they are collected in integrated vineyards in spring.

On the other hand, consuming dandelion from IPM vineyards could be potentially dangerous, especially when it is collected in a period when different pesticides are used in vineyards (from April to July). Namely, in every sample, more than one type of pesticide residue was detected, in some cases (4 out of 10) even with concentrations above MRL. In spite of the fact that residues exceeded MRL, dandelion was safe for consumers in terms of short-term risk assessment. Even though, in summer, collecting dandelions in integrated vineyards should be rare, as at that time the most intensive spraying of vines is carried out, and also plants growing underneath and in close vicinity seem to be influenced.

In autumn, no single PR was identified in dandelion leaves. It seems that dandelion leaves can be collected as well after harvest, at least as far as our study is concerned. But broader sampling with all commonly used pesticides in vineyards would be appreciated.

## Figures and Tables

**Table 1:** Active substances sought, MRM transitions, dwell time, fragmentor, and collision energy (CE) for active substances analyzed with GC-MS/MS and LC-MS/MS.

Active Substance	Instrument	Retention Time [min]	MRM Transitions	Dwell time [ms]	Fragmentor (V)	CE [V]
Boscalid	GC-MS/MS	34.3	342 → 112	148.9	/	30
			140 → 112			30
Difenoconazole	LC-MS/MS	12.8	406.1 → 337	200.0	107	197
			406.1 → 337			284
Dimethomorph	LC-MS/MS	10.6	388.1 → 301	200.0	142	20
		10.9	388.1 → 165			36
Kresoxim-methyl	GC-MS/MS	28.9	206 → 131	149.2	/	30
			206 → 116			30
Mandipropamid	LC-MS/MS	10.7	412.1 → 356.1	200.0	142	20
			412.1 → 328.1			36
Metalaxyl-M	GC-MS/MS	14.4	206 → 132	199.3	/	35
			206 → 105			35
Tebuconazole	GC-MS/MS	30.9	250 → 125	99.1	/	30
			250 → 70			30
Triadimenol	GC-MS/MS	20.6	168 → 70	82.5	/	40
		21.4	128 → 100			30

**Table 2:** Limit of quantification (LOQ), linearity range, square of correlation coefficient (R^2^), recovery, relative standard deviation (RSD) and number of replicates (N. rep.) for RSD calculation, uncertainty of repeatability (Ur) and uncertainty of reproducibility (U_R_) of multiresidue method for common dandelion (*Taraxacum officinale* agg.).

Active Substance	LOQ [mg/kg]	Linearity Range [mg/kg]	R^2^	Recovery [%]	RSD [%]	N. rep.	U_r_ [mg/kg]	U_r_ [%]	U_R_ [mg/kg]	U_R_ [%]
Boscalid	0.005	0.005–0.1	0.995	76.8	16.4	20	0.0005	10.0	0.0015	30.0
Difenoconazole	0.005	0.005–0.1	0.995	75.2	6.9	20	0.0003	7.4	0.0006	11.9
Dimethomorph	0.005	0.005–0.1	0.996	78.2	5.9	20	0.0003	6.7	0.0005	8.7
Kresoxim-methyl	0.005	0.005–0.1	0.993	80.1	13.5	20	0.0005	10.0	0.0013	26.0
Mandipropamid	0.005	0.005–0.1	0.999	80.4	7.9	20	0.0004	9.4	0.0007	17.9
Metalaxyl-M	0.005	0.005–0.1	0.993	85.0	12.3	20	0.0004	8.0	0.0012	24.0
Tebuconazole	0.005	0.005–0.1	0.995	71.5	15.2	20	0.0006	12.0	0.0013	26.0
Triadimenol	0.005	0.005–0.1	0.995	82.6	17.7	20	0.0010	20.0	0.0017	34.0

**Table 3:** Number of samples (*n*), sample portions (%), and range (mg/kg) of selected active substances found in leaves of common dandelion (*Taraxacum officinale* agg.) from vineyards with integrated pest management in three sampling periods.

	Spring Sampling			Summer Sampling			Autumn Sampling		
Active Substance	*n*	Portion [%]	Range [mg/kg]	*n*	Portion [%]	Range [mg/kg]	*n*	Portion [%]	Range [mg/kg]
Boscalid	0	0	/	10	100	0.005–0.254	0	0	/
Difenoconazole	0	0	/	4	40	0.095–0.627	0	0	/
Dimethomorph	0	0	/	1	10	0.005–0.005	0	0	/
Kresoxim-methyl	0	0	/	6	60	0.007–0.029	0	0	/
Mandipropamid	0	0	/	6	60	0.013–0.024	0	0	/
Metalaxyl-M	0	0	/	4	40	0.005–0.007	0	0	/
Tebuconazole	1	10	0.005	8	80	0.008–0.028	0	0	/
Triadimenol	0	0	/	0	0	/	0	0	/

**Table 4:** Limit of quantification (LOQ), maximum residue levels (MRLs*), and sample portions under limit of quantification (< LOQ), under MRL (≤ MRL), and above MRL (>MRL) of selected active substances found in leaves of common dandelion (*Taraxacum officinale* agg.) from vineyards with integrated pest management in three sampling periods. *MRL values for dandelion in group 255,000 [45] as determined by EU legislation [76].

		Spring Sampling			Summer Sampling			Autumn Sampling		
**Active** **Substance**	**MRL [mg/kg]**	**Sample** **Portion < LOQ [%]**	Sample Portion ≤ MRL [%]	Sample Portion > MRL [%]	Sample Portion < LOQ [%]	Sample Portion ≤ MRL [%]	Sample Portion > MRL [%]	Sample Portion < LOQ [%]	Sample Portion ≤ MRL [%]	Sample Portion > MRL [%]
Boscalid	7	100	0	0	0	100	0	100	0	0
Difenoconazole	4	100	0	0	60	40	0	100	0	0
Dimethomorph	0.05	100	0	0	90	10	0	100	0	0
Kresoxim-methyl	0.01	100	0	0	40	20	40	100	0	0
Mandipropamid	0.15	100	0	0	40	60	0	100	0	0
Metalaxyl-M	0.4	100	0	0	60	40	0	100	0	0
Tebuconazole	0.15	90	10	0	20	80	0	100	0	0
Triadimenol	0.01	100	0	0	100	0	0	100	0	0

## Data Availability

The original contributions presented in this study are included in the article/Supplementary Materials. Further inquiries can be directed to the corresponding author.

## Supplementary Materials

The following supporting information can be downloaded at: https://www.mdpi.com/article/10.3390/foods14040684/s1, Table S1: Fungicides used in vineyards under Integrated Pest and Disease Management in Goriška brda winegrowing district in a year before sampling (vintage 2021); Table S2: Fungicides used in vineyards under Integrated Pest and Disease Management in Goriška brda winegrowing district in a year of sampling (vintage 2022); Table S3: Analyzed active substances, its MRLs, limits of quantification (LOQ) and levels (mg/kg) in leaves of common dandelion (*Taraxacum officinale* agg.) from integrated (I), organic (E) and biodynamic (B) vineyards in three sampling periods (V1: spring sampling, V2: summer sampling when the plants regrew after the mulching, V3: autumn sampling when the plants regrew again after the last mulching).

## Abbreviations

The following abbreviations are used in this manuscript:

PR	Pesticide residues
IPM	Integrated pest and disease management
ORG	Organic
BIOD	Biodynamic
LOQ	Limit of quantification
MRL	Maximum residue limit
ADI	Acceptable Daily Intake
