# Supplementary material for: Is Common Dandelion (Taraxacum officinale agg.) Foraged for Food in Vineyards Pesticide Residues Free?
Source: Foods. 2025 Feb 17;14(4):684. doi: 10.3390/foods14040684 (PMC11854272; doi:10.3390/foods14040684)
Supplement: Supplementary file 1 [file foods-14-00684-s001.zip › Skubic et al_Supplementary Table S1, Table S2_new.pdf]

Table S1: Fungicides used in vineyards under Integrated Pest and Disease Management in Goriška brda winegrowing district in a year before sampling (vintage 2021).

Number of spraying	Date of application	Name of the product	Active substance	Dosage (kg/ha or L/ha)
1	3.05.2021	Manfil 75 WG	mancozeb	2.0 kg/ha
		Cosan	sulphur	7.0 kg/ha
2	10.05.2021	Manfil 75 WG	mancozeb	2.0 kg/ha
		Cosan	sulphur	7.0 kg/ha
3	17.05.2021	Folpan 80 WDG	folpet	1.25 kg/ha
		Tebusha 25% EW	tebuconazole	0.4 L/ha
4	24.05.2021	Manfil 75 WG	mancozeb	2.0 kg/ha
		Cosan	sulphur	4.0 kg/ha
5	31.05.2021	Folpan Gold	folpet + metalaxyl-M	2.5 kg/ha
		Tebusha 25% EW	tebuconazole	0.4 L/ha
6	9.06.2021	Sfinga Extra WDG	dimethomorph + folpet	2.0 kg/ha
		Vivando	metrafenone	0.2 L/ha
7	19.06.2021	Pergado F	mandipropamid + folpet	2.5 kg/ha
		Vivando	metrafenone	0.2 L/ha
8	28.06.2021	Fortuna Gold	cymoxanil + mancozeb	2.5 kg/ha
		Spirox D	difenoconazole	0.5 L/ha
9	7.07.2021	Orvego	ametoctradin + dimethomorph	0.8 L/ha
		Collis	boscalid + kresoxim-methyl	0.4 L/ha
10	16.07.2021	Reboot	cymoxanil + zoxamide	0.4 kg/ha
		Luna Experience	fluopyram + tebuconazole	0.4 L/ha
11	23.07.2021	Folpan 80 WDG	folpet	1.25 kg/ha
		Kumulus DF	sulphur	4.0 kg/ha
12	30.07.2021	Cuprovin 50	copper	2.0 kg/ha
13	10.08.2021	Cuprovin 50	copper	0.4 L/ha
		Kumulus DF	sulphur	2.0 kg/ha

Table S2: Fungicides used in vineyards under Integrated Pest and Disease Management in Goriška brda winegrowing district in a year of sampling (vintage 2022).

**Number of spraying**	**Date of application**	**Name of the product**	**Active substance**	**Dosage (kg/ha or L/ha)**
1	25.03.2022	Azumo	sulphur	3.5 kg/ha
2	12.04.2022	Polyram DF	metiram	2.4 kg/ha
		Azumo	sulphur	3.5 kg/ha
3	26.04.2022	Folpan 80 WDG	folpet	1.25 kg/ha
		Karathane Gold EC	meptyldinocap	0.5 L/ha
4	6.05.2022	Folpan 80 WDG	folpet	1.25 kg/ha
		Azumo	sulphur	3.5 kg/ha
		Tebusha 25% EW	tebuconazole	0.4 L/ha
5	16.05.2022	Reboot	cymoxanil + zoxamide	0.4 kg/ha
		Domark 100 EC	tetraconazole	0.3 L/ha
6	26.05.2022	Azumo	sulphur	5.0 kg/ha
		Pergado F	mandipropamid + folpet	2.5 kg/ha
7	8.06.2022	Revyona	mefentrifluconazole	2.0 L/ha
		Orvego	ametoctradin + dimethomorph	0.8 L/ha
8	20.06.2022	Collis	boscalid + kresoxim-methyl	0.4 L/ha
		Dynali	cyflufenamid + difenoconazole	0.6 L/ha
9	25.06.2022	Sivanto Prime	flupspiroyradifurone	0.5 L/ha
		Azumo	sulphur	7.0 kg/ha
10	3.07.2022	Folpan Gold	folpet + metalaxyl-M	2.0 kg/ha
		Azumo	sulphur	3.5 kg/ha
11	15.07.2022	Folpan 80 WDG	folpet	1.25 kg/ha
		Azumo	sulphur	3.5 kg/ha
12	22.07.2022	Prosper CS 300	spiroxamine	1.0 L/ha
		Azumo	sulphur	7.0 kg/ha
13	22.07.2022	Badge WG	copper substances - copper hydroxide	1.25 kg/ha
